# Detection of myeloma-associated osteolytic bone lesions with energy-integrating and photon-counting detector CT

**DOI:** 10.1007/s00117-024-01344-7

**Published:** 2024-07-17

**Authors:** Martin Grözinger, Markus Wennmann, Stefan Sawall, Eckhard Wehrse, Sam Sedaghat, Christian Neelsen, Fabian Bauer, Hartmut Goldschmidt, Vivienn Weru, Christian H. Ziener, Annette Kopp-Schneider, Heinz-Peter Schlemmer, Lukas T. Rotkopf

**Affiliations:** 1 Division of Radiology, German Cancer Research Center, Im Neuenheimer Feld 280, 69120 Heidelberg, Baden-Württemberg Germany; 2 Division of X-Ray Imaging and CT, German Cancer Research Center, Im Neuenheimer Feld 280, 69120 Heidelberg, Baden-Württemberg Germany; 3 Department of Diagnostic and Interventional Radiology, University Hospital of Heidelberg, Im Neuenheimer Feld 410, 69120 Heidelberg, Germany; 4 Department of Medicine V, Multiple Myeloma Section, University Hospital Heidelberg, Im Neuenheimer Feld 410, 69120 Heidelberg, Germany; 5 Division of Biostatistics, German Cancer Research Center, Im Neuenheimer Feld 280, 69120 Heidelberg, Baden-Württemberg Germany; 6 Medical Faculty, Ruprecht-Karls-University Heidelberg, Im Neuenheimer Feld 672, 69120 Heidelberg, Baden-Württemberg Germany

**Keywords:** Multiple myeloma, Photon-counting CT, Osteolytic lesions, Lesion detection, Image quality, Multiples Myelom, Photonenzählende Computertomographie, Osteolytische Läsionen, Läsionserkennung, Bildqualität

## Abstract

**Background:**

A recent innovation in computed tomography (CT) imaging has been the introduction of photon-counting detector CT (PCD-CT) systems, which are able to register the number and the energy level of incoming x‑ray photons and have smaller detector elements compared with conventional CT scanners that operate with energy-integrating detectors (EID-CT).

**Objectives:**

The study aimed to evaluate the potential benefits of a novel, non-CE certified PCD-CT in detecting myeloma-associated osteolytic bone lesions (OL) compared with a state-of-the-art EID-CT.

**Materials and methods:**

Nine patients with multiple myeloma stage III (according to Durie and Salmon) underwent magnetic resonance imaging (MRI), EID-CT, and PCD-CT of the lower lumbar spine and pelvis. The PCD-CT and EID-CT images of all myeloma lesions that were visible in clinical MRI scans were reviewed by three radiologists for corresponding OL. Additionally, the visualization of destructions to cancellous or cortical bone, and trabecular structures, was compared between PCD-CT and EID-CT.

**Results:**

Readers detected 21% more OL in PCD-CT than in EID-CT images (138 vs. 109; *p* < 0.0001). The sensitivity advantage of PCD-CT in lesion detection increased with decreasing lesion size. The visualization quality of cancellous and cortical destructions as well as of trabecular structures was rated higher by all three readers in PCD-CT images (mean image quality improvements for PCD-CT over EID-CT were +0.45 for cancellous and +0.13 for cortical destructions).

**Conclusions:**

For myeloma-associated OL, PCD-CT demonstrated significantly higher sensitivity, especially with small size. Visualization of bone tissue and lesions was considered significantly better in PCD-CT than in EID-CT. This implies that PCD-CT scanners could potentially be used in the early detection of myeloma-associated bone lesions.

**Graphic abstract:**

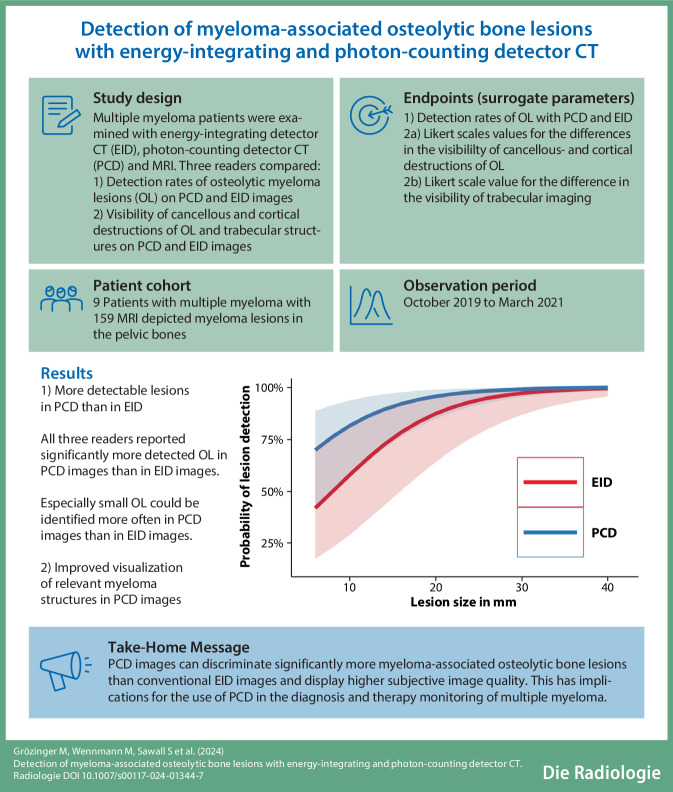

**Supplementary Information:**

The online version of this article (10.1007/s00117-024-01344-7) contains supplementary tables, which are available to authorized users.

## Abstract (de)

**Hintergrund:**

Eine aktuelle Innovation in der Bildgebung mittels Computertomographie (CT) stellt die Einführung photonenzählender Detektor-CT(PCD-CT)-Systeme dar, welche die Anzahl und das Energieniveau einfallender Röntgenphotonen zu registrieren vermögen und kleinere Detektorelemente als konventionelle CT-Geräte aufweisen, die mit energieintegrierenden Detektoren arbeiten (EID-CT).

**Ziel:**

Ziel der vorliegenden Studie war es, die potenziellen Vorteile einer neuartigen, nicht-CE-zertifizierten PCD-CT bei der Erkennung myelomassoziierter osteolytischer Knochenläsionen (OL) im Vergleich zu EID-CT auf dem Stand der Technik zu ermitteln.

**Material und Methoden:**

Bei 9 Patienten mit multiplem Myelom im Stadium III (nach Durie und Salmon) wurde eine Magnetresonanztomographie (MRT), EID-CT und PCD-CT der unteren Lendenwirbelsäule und des Beckens durchgeführt. Die PCD-CT- und EID-CT-Aufnahmen aller Myelomläsionen, die auf den klinischen MRT-Bildern sichtbar waren, wurden von 3 Radiologen in Bezug auf entsprechende OL beurteilt. Zusätzlich wurde die Visualisierung von Destruktionen der Spongiosa, der Kortikalis und trabekulärer Strukturen zwischen PCD-CT und EID-CT verglichen.

**Ergebnisse:**

Es wurden 21% mehr OL in PCD-CT- als in EID-CT-Aufnahmen erkannt (138 vs. 109; *p* < 0,0001). Der Vorteil der PCD-CT hinsichtlich der Sensitivität bei der Läsionserkennung nahm mit abnehmender Läsionsgröße zu. Die Visualisierungsqualität spongiöser und kortikaler Destruktionen sowie von trabekulären Strukturen wurden von allen 3 Untersuchern bei den PCD-CT-Aufnahmen als höher beurteilt (die durchschnittliche Verbesserung der Bildqualität bei PCD-CT gegenüber EID-CT betrug +0,45 für spongiöse und +0,13 für kortikale Destruktionen).

**Schlussfolgerung:**

Für myelomassoziierte OL zeigte die PCD-CT eine signifikant höhere Sensitivität, insbesondere bei geringer Größe. Hinsichtlich der Visualisierung von Knochengewebe und -läsionen wurde die PCD-CT signifikant besser als die EID-CT beurteilt. Das bedeutet, dass PCD-CT-Geräte möglicherweise zur Früherkennung von myelomassoziierten Knochenläsionen eingesetzt werden könnten.

## Background

Imaging plays a major role in the diagnosis, risk stratification, and therapy response assessment of multiple myeloma [1–5]. Whole-body magnetic resonance tomography (MRI) and positron emission tomography (PET) enable imaging of the bone marrow itself and may therefore depict focal lesions in asymptomatic precursor states before frank destruction of mineralized bone [6–10]. Computed tomography (CT) can depict the destruction of mineralized bone, which is of high importance, given that most patients progressing from smoldering myeloma (SMM) to multiple myeloma (MM) show osteolytic lesions [10] and that around 90% of MM patients will eventually suffer from osteolytic lesions [11]. The development of osteolytic bone disease is a major topic in current myeloma research [12]. Current guidelines further acknowledge that whenever MRI or PET is unavailable, CT is the primary imaging modality, as is the case in many countries worldwide [2].

A recent innovation in CT is the introduction of photon-counting detector computed tomography (PCD-CT) systems, which register the number and the energy level of incoming x‑ray photons using much smaller detector elements in comparison with conventional energy-integrating computed tomography detectors (EID; [13, 14]). In contrast to EIDs, which depend on scintillator elements, PCDs consist of semiconductor materials such as cadmium-telluride. The absorption of an x‑ray photon results in the formation of a charge cloud that is transported to electrodes, where the desired signal is being processed, using attached high-speed electronics [14, 15]. Dedicated high-resolution (HR) reconstruction kernels are used to reconstruct images with smaller voxel sizes [15]. Therefore, PCD-CT has several technical advantages over EID-CT: increased spatial resolution [16], less image noise [17], higher dose efficiency [18], and intrinsic dual- or multi-energy acquisition [19].

Initial studies on myeloma imaging with PCD-CT have already shown advantages in image quality, particularly regarding the delineation of trabeculae, cortical bone, and myeloma-associated osteolytic lesions [20–23]. Additionally, the first proof-of-concept studies reported that more myeloma-associated osteolytic bone lesions were visible on PCD-CT than on EID-CT images [22, 23].

## Methods

### Patient population

**Fig. 1:**
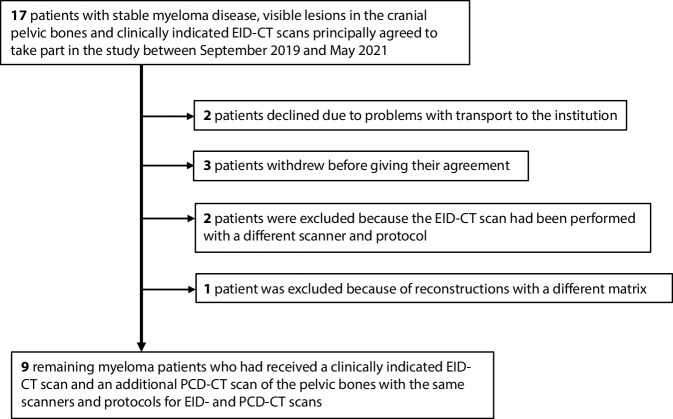
Flowchart of patients included in this study

Patient recruitment started after approval from the local institutional review board and the radiation protection authority. A flowchart of the patient selection process and detailed inclusion/exclusion information can be found in Fig. 1. Before study inclusion, each patient gave written informed consent. Due to the preclinical nature of our study and the dose exposure from the additional scan using a non-CE-certified prototype PCD-CT scanner, the sample size was pre-set to a maximum of 20 myeloma patients, and the z‑range of the scan was limited as recommended by the ethics committee and the radiation protection authority. In the end, nine patients with stage III (according to Durie and Salmon) myeloma (time since diagnosis 30.6 ± 28.4 months) and with known focal lesions were included in this prospectively planned study. All individuals had received induction therapy and high-dose chemotherapy with autologous stem cell transplantation and, at the time of image acquisition, were under maintenance therapy without progression. Additional inclusion criteria were an age of at least 18 years and legal competence. Patient age ranged from 48 to 75 years. Six patients were male and three were female.

All patients underwent a clinically indicated EID-CT scan and an MRI examination. The additional PCD-CT scan covered the upper parts of the pelvic bones and the lower lumbar spine, using an experimental prototype PCD-CT scanner (SOMATOM CounT, Siemens Healthineers, Forchheim, Germany). The PCD-CT examinations were acquired within a mean time interval of 1.1 months (range: same day to 5 months) to the previously acquired or planned EID-CT scan. A detailed overview of the time intervals between the MRI, EID-CT, and PCD-CT scans is provided in Supplemental Table 1.

### CT image acquisition and reconstruction

The EID-CT scans were performed on a state-of-the-art spectral CT scanner (IQon Spectral CT, Philips Healthcare, Best, The Netherlands), as shown in Table 1, using standard clinical acquisition parameters and a manufacturer-specific YA bone kernel. The tube voltage applied was 120 kV without an additional tin filtration technique in the EID- and PCD-CT scans. For PCD-CT scans, a prospectively defined tube current–time product of 300 mAs was applied to enable sufficient image quality in patients with larger girths, based on the experiences from phantom measurements. Due to automatic dose reduction in EID-CT, the mean applied current–time product over the pelvic bones was lower in the EID-CT than in PCD-CT scans (EID-CT: 180 ± 64 mAs, range: 88–550 mAs). The PCD-CT images were acquired using a specific ultra-high resolution (UHR) mode with a 2 × 2 subdetector binning readout technique and specific bone kernels (U70) to reconstruct axial slices with voxel sizes of 0.53 mm × 0.53 mm (in-plane) × 1.0 mm (z-direction).

**Table 1:** Acquisition and reconstruction parameters of EID-CT and PCD-CT scans

CT system	EID-CT	PCD-CT
Number of patients	9	9
Scanner platform	Philips Spectral CT 7500	Siemens Somatom CounT
Detector type	Spectral EID	PCD
Collimation	128 × 0.6 mm	32 × 0.3 mm
Tube voltage	120 kV	120 kV
Effective tube current Field of view	180 mAs (range: 88–550 mAs) 400 mm	300 mAs 275 mm
Kernel/matrix size	YA/512 × 512	U70f/512 × 512
Slice thickness Increment	2 mm 1.5 mm	1 mm 0.5 mm
In-plane pixel size	0.8 × 0.8 mm^2^	0.5 × 0.5 mm^2^

All MR images were acquired with standard sequence protocols on two different scanner systems of the same vendor (Siemens Healthineers, Erlangen, Germany): 1.5 T Siemens Magnetom Aera and 1.5 T Siemens Magnetom Avanto fit. The examinations included T1- and T2- as well as diffusion-weighted images and in some cases post-contrast sequences. For all patients, axial sequences in either T1-weighting or diffusion-weighting were available.

### Image analysis

**Fig. 2:**
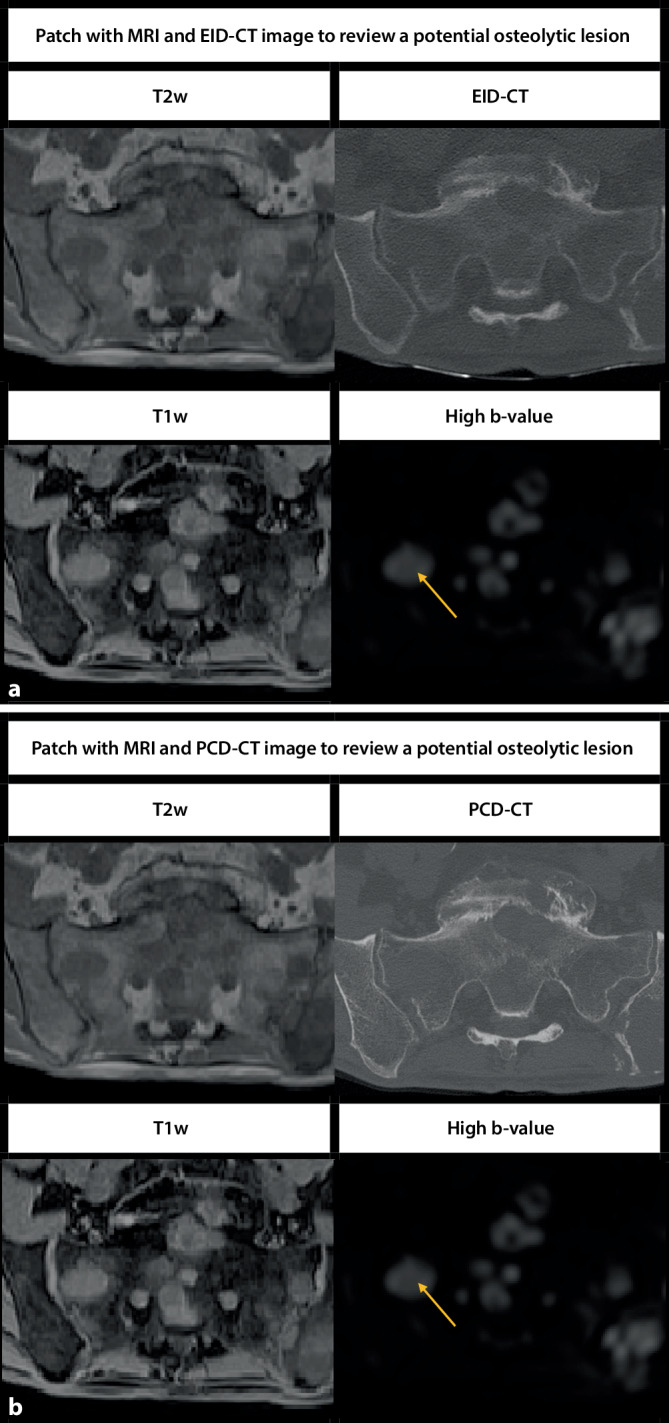
Visibility of lytic bone lesions in EID-CT (**a**) and PCD-CT (**b**). **a** *Upper patch* (2 × 2): axial T2-weighted image in the *upper left quadrant*, EID-CT slice in the upper right quadrant, T1-weighted image in the *lower left quadrant*, and DWI with high b‑value in the *lower right quadrant*. **b** *Lower patch* (2 × 2): axial T2-weighted sequence in the *upper left quadrant*, PCD-CT slice in the upper right quadrant, T1-weighted sequence in the *lower left quadrant*, and DWI with high b‑value in the *lower right quadrant*

In the first step, all focal myeloma lesions in the pelvic bones were identified using the axial MRI sequences as the reference standard. Secondly, each lesion detected on axial MR images was matched to corresponding EID-CT and PCD-CT images. Two batches of images, as exemplarily shown in Fig. 2, were created using the software ITK Snap [24] to compare the visualization of the lesions between EID-CT and PCD-CT images. Afterward, randomly assigned images from PCD-CT or EID-CT scans were manually assessed by a team of three readers blinded to the image type. Two readers were residents with 3 and 4 years of experience, respectively, and one reader was a board-certified radiologist with 7 years of experience. All readers reviewed all images individually and assessed whether the CT image showed a corresponding osteolytic lesion (OL). After this, the batches were shown to the readers again in the right order, always with the PCD-CT image following the EID-CT image. This time, readers were asked to assess, on three-point scales, whether the visualization of cancellous and cortical bone destruction was inferior, equal, or superior on the PCD-CT image. Finally, readers assessed differences in the visibility of trabecular structures between PCD-CT and EID-CT images on a five-point rating scale.

### Statistical analysis

Statistical differences in lesion detection rates between PCD-CT and EID-CT images were assessed using a logistical mixed-effects model adjusting for lesion size, localization, patient identity, and reader identity as random effects. A linear mixed-effects model on the log-transformed lesion size was fit to investigate whether localization influenced the lesion size, including patient and reader identity as random effects. Pairwise comparisons of the different localizations were performed from the mixed-effects model, with *p *values adjusted for multiple testing using the Holm method. All values of *p* < 0.05 were considered statistically significant. Statistical analysis was performed using R (version 4.0.3, R Core Team, Vienna, Austria).

## Results

### Number and size of the examined lesions

Among the nine patients, 159 MRI-visible myeloma lesions could be identified in the lower lumbar spine and the cranial pelvic bones. The average number of lesions per patient was 18 ± 20. The mean short-axis lesion size was 9.1 ± 5.0 mm. The majority of lesions were < 10 mm (107, 67%). An overview of the number of lesions and the ranges of lesion size per patient can be found in Supplemental Table 2. Lesions were located in the cranial parts of both pelvic bones (left side: 39, right side: 22), the sacral bone (86), and the caudal parts of the 5th lumbar vertebra (12). Pairwise comparisons of the various localizations showed that lesion sizes within the bones did not differ significantly.

### Sensitivity of lesion detection

**Fig. 3:**
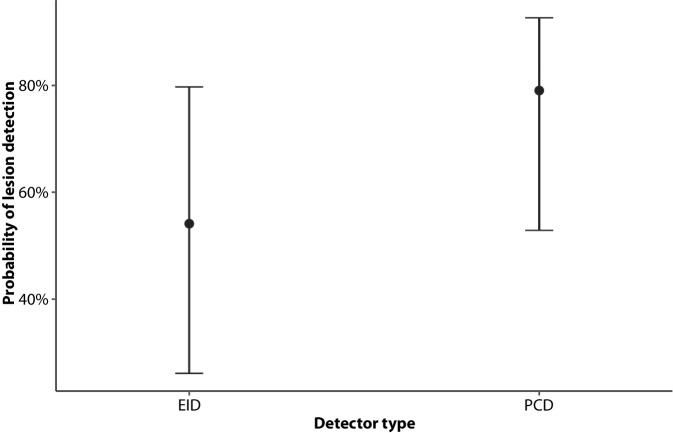
Predicted probability of lesion detection for both PCD-CT and EID-CT of a logistical mixed-effects model adjusting for lesion size, localization, patient identity, and reader identity as random effects

Each of the three readers detected significantly more OL in PCD-CT than in EID-CT images. Differences between scanner types were comparable between all three readers: reader one (PCD: 140 OL, EID: 106 OL, difference +24%); reader two (PCD: 96 OL, EID: 68 OL, difference +29%); and reader three (PCD: 147 OL, EID: 120 OL, difference +18%; for each reader individually *p* < 0.01). The majority of readers (at least two of three) found significantly more OL in PCD images (138 OL, 87%) than in EID images (109 OL, 69%), difference: 29 OL (21%) (*p* < 0.0001), resulting in different predicted probabilities in detection rates (Fig. 3).

### Sensitivity in lesion detection and lesion size

**Fig. 4:**
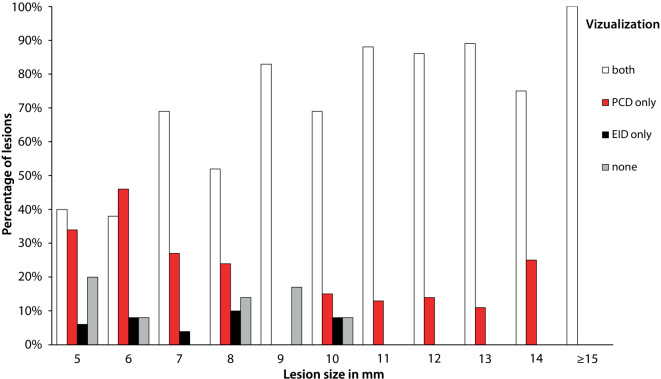
Percentage of lesions visible in images of both CT scanners, only in PCD-CT images, only in EID-CT images, and in neither image—depending on lesion size

**Fig. 5:**
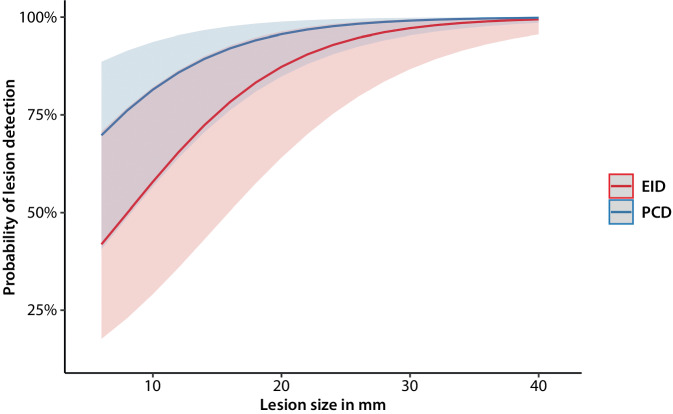
Predicted probability of lesion detection for PCD-CT and EID-CT depending on lesion size. The superiority of PCD-CT over EID-CT increased with decreasing lesion size. *Solid lines*: mean predicted probabilities to identify a lesion

With decreasing lesion size, the rate of lesions that showed no OL in neither one of the CT images increased, and the rates of lesions that could solely be detected in PCD-CT images, or in EID-CT images also increased. The rate of lesions detected in both CT images decreased with decreasing lesion size (Table 2 and Fig. 4). In a combined model including detector type and lesion size controlled for localization, both parameters were significant (*p* < 0.0001), indicating that for small lesions, the probability of being visible was higher in PCD-CT images than in EID-CT images (Table 2 and Fig. 5).

**Table 2:** Number and percentage of lesions stratified by lesion size that showed either no corresponding OL, OL only in EID-CT, OL only in PCD-CT, or OL in both CT images

Lesion size in mm	Total no. of lesions	Visibility of lesions depending on their size				Visibility of lesions depending on their size in %				Number of detected lesions		Percentage of detected lesions in %	
		Never	EID-CT	PCD-CT	Both	Never	EID-CT	PCD-CT	Both	EID-CT	PCD-CT	EID-CT	PCD-CT
5	35	7	2	12	14	20	6%	34%	40	16	26	46	74
6	13	1	1	6	5	8	8	46	38	6	11	46	85
7	26	0	1	7	18	0	4	27	69	19	25	73	96
8	21	3	2	5	11	14	10	24	52	13	16	62	76
9	12	2	0	0	10	17	0	0	83	10	10	83	83
10	13	1	1	2	9	8	8	15	69	10	11	77	85
11	8	0	0	1	7	0	0	13	88	7	8	88	100
12	7	0	0	1	6	0	0	14	86	6	7	86	100
13	9	0	0	1	8	0	0	11	89	8	9	89	100
14	4	0	0	1	3	0	0	25	75	3	4	75	100
≥ 15	11	0	0	0	11	0	0	0	100	0	0	0	0

### Differences in subjective image quality

All three readers unanimously reported improvements in the visualization of cancellous and cortical bone destructions in PCD-CT over EID-CT, as shown in Table 3. Overall mean image quality improvement for PCD-CT over EID-CT was +0.45 for cancellous bone and +0.13 for cortical bone destructions. All three readers reported improvements in the visualization of trabecular structures (Table 4).

**Table 3:** Visualization of cancellous and cortical bone destructions by osteolytic lesions

	Image quality improvements of cancellous destructions			Image quality improvements of cortical destructions		
	Reader 1	Reader 2	Reader 3	Reader 1	Reader 2	Reader 3
Mean scale value	+0.52	+0.20	+0.64	+0.24	+0.01	+0.13
SD	0.67	0.49	0.59	0.46	0.19	0.41

Mean values and standard deviations (SD) are based on reader-specific scoring of image quality on a three-point rating scale. Improvement in PCD-CT is reported in relation to EID-CT

**Table 4:** Visualization of trabecular structures using PCD-CT in comparison with EID-CT

	Reader 1	Reader 2	Reader 3	Overall
Mean scale value	+2.00	+1.98	+1.00	1.66
SD	0.00	0.14	0.00	0.05

Mean values and standard deviations (SD) are based on reader-specific scoring of image quality on a five-point rating scale. Improvement in PCD-CT is reported in relation to EID-CT

## Discussion

In this prospective proof-of-concept study, we investigated the detection of osseous myeloma lesions using a prototype PCD-CT. First, we compared the detection rates of MRI-confirmed lesions with those from CT, revealing a significant advantage of PCD-CT that further increased as lesion size decreased. Second, readers rated the subjective image quality and lesion depiction quality considerably higher in PCD-CT than in EID-CT.

These results imply that PCD-CT may have benefits in the early detection of myeloma-associated osteolytic bone lesions, while potentially narrowing the current sensitivity gap between CT and MRI [25, 26]. Due to smaller detector elements in PCD, reliable detection of osteolytic lesions below the currently applied minimum size of 5 mm, according to slim-CRAB criteria as defined by the IMWG, might be possible.

The improved visualization of destructions to cancellous and cortical bone as well as of trabecular structures is in concordance with prior PCD-CT studies with myeloma patients that reported higher overall image quality, detectability, and sharpness in the delineation of lytic bone lesions [21, 27] and improvements in the depiction of lytic lesions, intramedullary lesions, fatty metamorphosis, and pathologic fractures [22]. Also, the reported improvements in the detection rates of OL in myeloma patients are in accordance with the results of previous studies. In an investigation by Baffour et al., 27 myeloma patients received whole-body EID-CT scans and corresponding PCD-CT scans. The authors reported that in 21 of the 27 scanned patients, the group detected at least one more OL in the PCD-CT scans with UHR mode and dedicated bone kernels (in their case B76) than in the EID-CT scans. In another recently published study by Schwartz et al., a superior detection rate of 79% (22 of 28 OL) in PCD-CT images in comparison with 64% (18 of 28 OL) in EID-CT images was observed [23]. In both of these studies, the applied radiation doses in PCD-CT scans were equal to or lower than the EID-CT scans.

### Limitations

Our study has limitations. First, different tube current–time products (and proportionally radiation doses) were applied in PCD-CT and EID-CT. Therefore, differences in lesion detection and image quality may not only arise from improvements in detector design but also from reduced noise due to higher radiation dose. In this preregistered proof-of-concept study, it was necessary to define the tube current–time product before the inclusion of the first patients. This important limitation must be addressed in further controlled studies using either matched doses or automatic dose modulation. Third, potentially favoring the EID-CT lesion detection rate, more noise might have been present in PCD-CT due to the lower slice thickness and similar matrix size of 512 × 512 based on the experiences of recent studies; hence the potential of the UHR mode in the PCD-CT could not be fully leveraged [28]. Fourth, the time interval between PCD-CT and EID-CT was heterogeneous. Finally, the number of included patients was rather small; however, ethical considerations did not allow for more extensive investigations until preliminary studies like this would ascertain the safety of the prototype scanner. Further studies may build upon the presented proof-of-concept data and include more patients.

## Conclusion

This study demonstrates that photon-counting detector computed tomography (PCD-CT) using sharp bone kernels and the ultra-high resolution mode can discriminate significantly more myeloma-associated osteolytic bone lesions than conventional energy-integrating detector (EID)-CT and display higher subjective image quality. This has potential implications for the use of PCD-CT in the diagnosis and therapy monitoring of multiple myeloma.

## Supplementary Information

Supplemental Table 1: Time intervals between MRI, EID-CT, and PCD-CT scans. Supplemental Table 2: Number of lesions and ranges of lesion size per patient.

## Data Availability

The data that support the findings of this study are available upon reasonable request from the corresponding author. The data are not publicly available due to privacy or ethical restrictions. However, a de-identified dataset, excluding any sensitive patient information, can be made available to qualified researchers after approval from the institutional review board and compliance with relevant data protection regulations. Requests for data access should be directed to: Dr. med. Lukas T. Rotkopf (l.rotkopf@dkfz-heidelberg.de) and will be subject to an evaluation process to ensure responsible and ethical use of the data.

## Abbreviations

CT: Computed tomography
DWI: Diffusion-weighted imaging
EID: Energy-integrating detector
MM: Multiple myeloma
MRI: Magnetic resonance imaging
MTF: Modulation transfer function
PCD: Photon-counting detector
SMM: Smoldering multiple myeloma

## Declarations

All procedures performed in studies involving human participants or on human tissue were in accordance with the ethical standards of the institutional and/or national research committee and with the 1975 Helsinki declaration and its later amendments or comparable ethical standards. Informed consent was obtained from all individual participants included in the study.

The supplement containing this article is not sponsored by industry.
