# Supplementary material for: Detection of myeloma-associated osteolytic bone lesions with energy-integrating and photon-counting detector CT
Source: Radiologie (Heidelb). 2024 Jul 17;64(Suppl 1):24–31. doi: 10.1007/s00117-024-01344-7 (PMC12662884; doi:10.1007/s00117-024-01344-7)
Supplement: Supplementary file 1 — Supplemental Table 1: Time intervals between MRI, EID-CT, and PCD-CT scans. Supplemental Table 2: Number of lesions and ranges of lesion size per patient. [file 117_2024_1344_MOESM1_ESM.docx]

**Supplemental Table 1:** Time intervals between MRI, EID-CT, and PCD-CT scans.

| **Patient no.** | **Date MRI** | **Date EID-CT** | **Date PCD-CT** | **Time between MRI and EID-CT in days** | **Time between MRI and PCD-CT in days** | **Time between EID-CT and PCD-CT in days** | **Time between MRI and EID-CT in months** | **Time between MRI and PCD-CT in months** | **Time between EID-CT and PCD-CT in months** |
| --- | --- | --- | --- | --- | --- | --- | --- | --- | --- |
| **1** | 27.01.21 | 17.11.20 | 03.02.21 | 71 | 7 | 78 | 2.3 | 0.2 | 2.6 |
| **2** | 23.07.20 | 28.08.20 | 14.08.20 | -36 | 22 | -14 | -1.2 | 0,7 | -0.5 |
| **3** | 14.02.20 | 17.05.19 | 10.10.19 | 273 | -127 | 146 | 9 | -4.2 | 4.8 |
| **4** | 18.02.21 | 08.02.21 | 18.02.21 | 10 | 0 | 10 | 0.3 | 0 | 0.3 |
| **5** | 30.09.20 | 28.08.20 | 30.09.20 | 33 | 0 | 33 | 1.1 | 0 | 1.1 |
| **6** | 29.01.21 | 03.03.21 | 15.02.21 | -33 | 17 | -16 | -1.1 | 0.6 | -0.5 |
| **7** | 02.09.20 | 02.09.20 | 02.09.20 | 0 | 0 | 0 | 0 | 0 | 0 |
| **8** | 05.11.21 | 02.10.20 | 06.11.20 | 399 | -364 | 35 | 13.1 | -11.9 | 1.1 |
| **9** | 01.10.20 | 10.09.20 | 01.10.20 | 21 | 0 | 21 | 0.7 | 0 | 0.7 |
| **Mean** |  |  |  | 82 | -49 | 33 | 2.7 | -1.6 | 1.1 |
| **SD** |  |  |  | 151 | 126 | 51 | 5.0 | 4.1 | 1.7 |

**Supplemental Table 2:** Number of lesions and ranges of lesion size per patient.

| **Patient no.** | **Sex** | **Age** | **No. of lesions per patient** | **Mean lesion size in mm** | **Minimum lesion size in mm** | **Maximum lesion size in mm** |
| --- | --- | --- | --- | --- | --- | --- |
| **1** | w | 69 | 1 | 6.0 | 6 | 6 |
| **2** | m | 63 | 22 | 6.6 | 5 | 12 |
| **3** | m | 59 | 2 | 15.5 | 14 | 17 |
| **4** | w | 75 | 57 | 9.4 | 5 | 27 |
| **5** | m | 50 | 44 | 8.9 | 5 | 31 |
| **6** | m | 71 | 1 | 25.0 | 25 | 25 |
| **7** | m | 53 | 15 | 7.9 | 5 | 41 |
| **8** | w | 48 | 4 | 6.5 | 5 | 9 |
| **9** | m | 65 | 13 | 12.6 | 5 | 41 |
